# A novel variant of the Calvin–Benson cycle bypassing fructose bisphosphate

**DOI:** 10.1038/s41598-022-07836-7

**Published:** 2022-03-16

**Authors:** Jun Ohta

**Affiliations:** Graduate School of Medicine, Dentistry and Pharmaceutical Sciences, Okayama University, 2-5-1 Shikatacho, Kita-ku, Okayama, 700-8558 Japan

**Keywords:** Photosynthesis, C3 photosynthesis, Biochemical networks

## Abstract

The Calvin–Benson cycle (CB cycle) is quantitatively the most important metabolic pathway for CO_2_ fixation. In the canonical CB cycle, fructose 6-phosphate (F6P), fructose 1,6-bisphosphate (FBP), sedoheptulose 7-phosphate (S7P), and sedoheptulose 1,7-bisphosphate (SBP) appear as essential intermediates, where F6P is formed from FBP by the fructose 1,6-bisphosphatase (FBPase) reaction, and S7P is formed from SBP by the sedoheptulose 1,7-bisphosphatase (SBPase) reaction. Although the involvement of SBP and SBPase in the canonical CB cycle is consistent with the reported dependency of photosynthetic carbon metabolism on SBPase, the involvement of FBP and FBPase is not completely consistent with the reported FBP- or FBPase-related findings such as, although with a diminished growth rate, an *Arabidopsis* mutant lacking FBPase grew photoautotrophically in soil. Here, we show a novel variant of the CB cycle involving SBP, SBPase, and transaldolase, but neither FBP nor FBPase. This novel variant, named the S7P-removing transaldolase variant, bypasses FBP. This variant explains the FBP- or FBPase-related findings more easily than the canonical CB cycle as well as the dependency of photosynthetic carbon metabolism on SBPase and further suggests that co-overexpression of SBPase and transaldolase can be a strategy for enhancing photosynthetic carbon metabolism, which is important for the global environment.

## Introduction

The Calvin–Benson cycle (CB cycle) is ‘quantitatively the most important CO_2_ fixation mechanism in the modern biosphere’^1^. The canonical CB cycle^2^ is described in most textbooks of biochemistry. In the canonical CB cycle, one molecule of glyceraldehyde 3-phosphate (GAP) is formed from 3 molecules of CO_2_ through a pathway composed of a total of 29 reactions from the 13 enzyme reactions shown in Table 1. Whereas the canonical CB cycle is so established, there are puzzling observations such as incomplete labeling of the CB cycle intermediates after ^14^CO_2_ or ^13^CO_2_ treatment of photosynthesizing leaves and induction of a high cyclic photosynthetic electron flow by a lack of FBPase^3–5^. These observations were reported together with 2 findings whose mechanism is difficult to understand. One of the 2 findings is that treatment of an illuminated *Arabidopsis* rosette with ^13^CO_2_ resulted in slower labeling of FBP than metabolites located further downstream of FBP in the canonical CB cycle, such as SBP, S7P, and ribulose 1,5-bisphosphate (RuBP)^3^. Another is that, although with a diminished growth rate, an *Arabidopsis* mutant lacking FBPase essential to the canonical CB cycle grew photoautotrophically in soil^4^. Direct interpretation of these FBP- or FBPase-related findings leads to the idea that the CO_2_ fixation pathway involving neither FBP formation catalyzed by aldolase nor FBPase reaction, where FBP is bypassed, may be functioning. This paper proposes a novel CO_2_ fixation pathway involving neither FBP formation catalyzed by aldolase nor FBPase reaction. This novel CO_2_ fixation pathway is a variant of the CB cycle where transaldolase is involved.

**Table 1:** List of reactions constituting the canonical Calvin–Benson cycle^a,b,c^.

Reaction number and name		Substrate		Product
1	Rubisco	CO_2_ + RuBP	→	2 PGA
2	PGA kinase	PGA	→	BPG
3	GAP dehydrogenase	BPG	→	GAP
4	Triose phosphate isomerase	GAP	→	DHAP
5	FBP aldolase	GAP + DHAP	→	FBP
6	FBPase	FBP	→	F6P
7	Transketolase	F6P + GAP	→	E4P + Xu5P
8	SBP aldolase	E4P + DHAP	→	SBP
9	SBPase	SBP	→	S7P
10	Transketolase	S7P + GAP	→	R5P + Xu5P
11	Isomerase	R5P	→	Ru5P
12	Epimerase	Xu5P	→	Ru5P
13	Phosphoribulokinase	Ru5P	→	RuBP

^a^Currency metabolites are not shown. Currency metabolites include metabolites such as ATP, ADP, NADPH, NADP^+^, H^+^, inorganic phosphate, and H_2_O.

^b^PGA kinase, 3-phosphoglycerate kinase; GAP dehydrogenase, glyceraldehyde 3-phosphate dehydrogenase; FBP aldolase, fructose 1,6-bisphosphate aldolase; FBPase, fructose 1,6-bisphosphatase; SBP aldolase, sedoheptulose 1,7-bisphosphate aldolase; SBPase, sedoheptulose 1,7-bisphosphatase; RuBP, ribulose 1,5-bisphosphate; PGA, 3-phosphoglycerate; BPG,1,3-bisphosphoglycerate; GAP, glyceraldehyde 3-phosphate; DHAP, dihydroxyacetone phosphate; FBP, fructose 1,6-bisphosphate; F6P, fructose 6-phosphate; E4P, erythrose 4-phosphate; Xu5P, xylulose 5-phosphate; SBP, sedoheptulose 1,7-bisphosphate; S7P, sedoheptulose 7-phosphate; R5P, ribose 5-phosphate; Ru5P, ribulose 5-phosphate.

^c^The FBP and SBP formation reactions catalyzed by aldolase are named FBP aldolase and SBP aldolase, respectively.

Transaldolase (EC 2.2.1.2) is an enzyme present in photosynthetic tissue^6,7^. For proteins assumed to be transalodolase proteins existing in chloroplasts, UniProtKB has more than 20 entries having the value ‘Protein inferred by homology’, which are from more than 20 species^8^. The proteins encoded by the genes from *Arabidopsis thaliana* indexed as *AT5G13420* and *AT1G12230* are assumed to function as transaldolases and have been shown to exist in chloroplasts by proteomic analysis of chloroplasts of *Arabidopsis thaliana*^9–11^. As described below, *AT5G13420*-encoded transaldolase has recently been reported to function in *Arabidopsis* chloroplasts^12^. According to the Genevestigator database, the transcripts of *AT5G13420* and *AT1G12230* are widely distributed in *Arabidopsis thaliana* tissues at medium to high expression levels^13^. Transaldolase can catalyze both the reaction forming S7P with GAP from erythrose 4-phosphate (E4P) and F6P and its reverse reaction removing S7P with GAP to make E4P and F6P. The variant of the CB cycle corresponding to the novel CO_2_ fixation pathway proposed here is called the S7P-removing transaldolase variant because it requires the S7P-removing action of transaldolase. On the other hand, another theoretical variant of the CB cycle with transaldolase involved was proposed previously^5,14^, which is called the S7P-forming transaldolase variant here because it requires the S7P-forming action of transaldolase.

## Results

**Figure 1:**
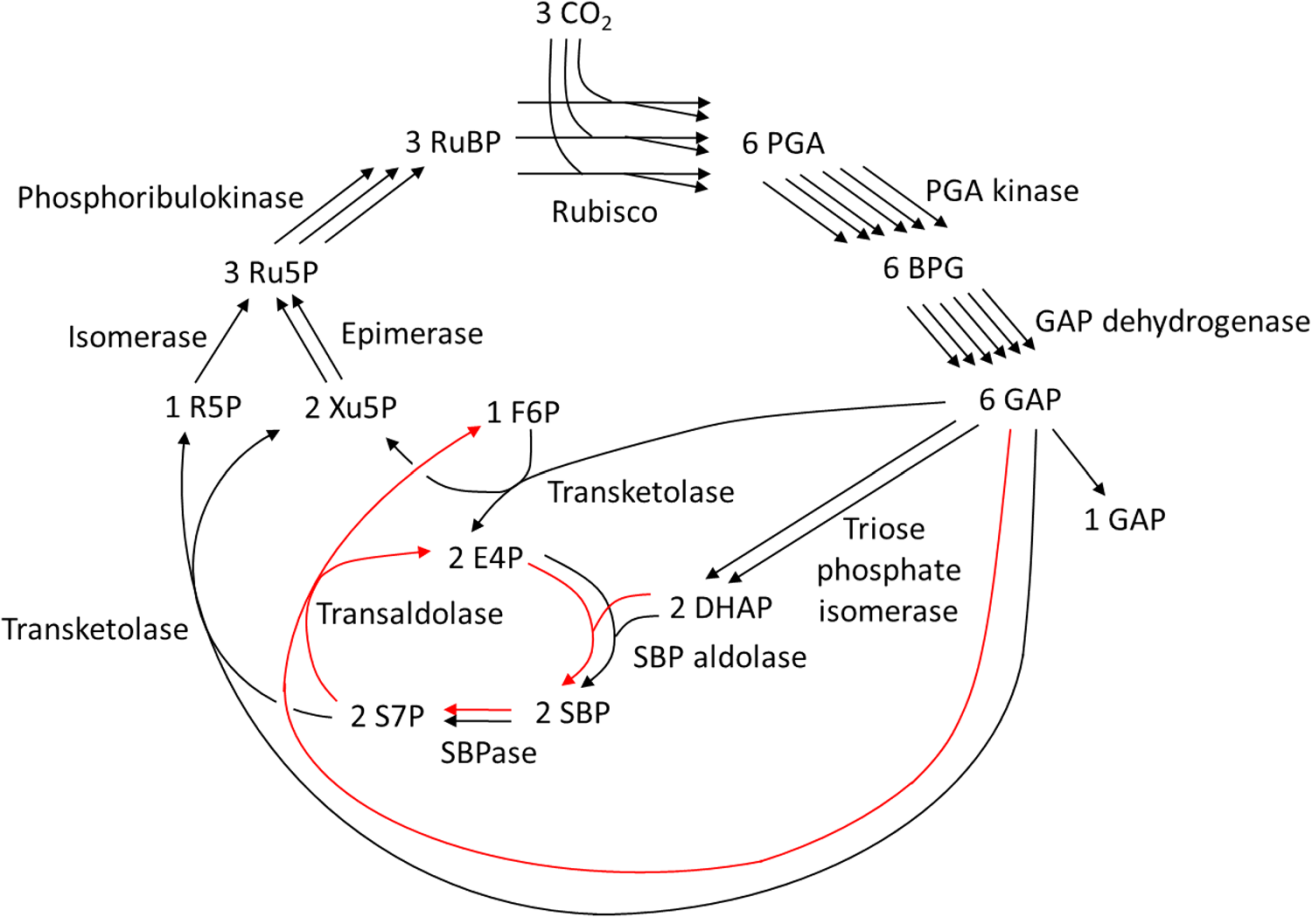
GAP formation from CO_2_ through the S7P-removing transaldolase variant of the Calvin–Benson cycle. The reactions corresponding to the red arrows appear in neither the canonical Calvin–Benson cycle nor the S7P-forming transaldolase variant. GAP and S7P indicate glyceraldehyde 3-phosphate and sedoheptulose 7-phosphate, respectively. For other abbreviations, please see Table 1. The number before each metabolite, such as 3 for CO_2_ and 6 for PGA, is equal to the number of arrows entering or exiting the metabolite. Currency metabolites are not shown. Currency metabolites include metabolites such as ATP, ADP, NADPH, NADP^+^, H^+^, inorganic phosphate, and H_2_O. In this paper, the FBP and SBP formation reactions catalyzed by aldolase are named FBP aldolase and SBP aldolase, respectively.

**Figure 2:**
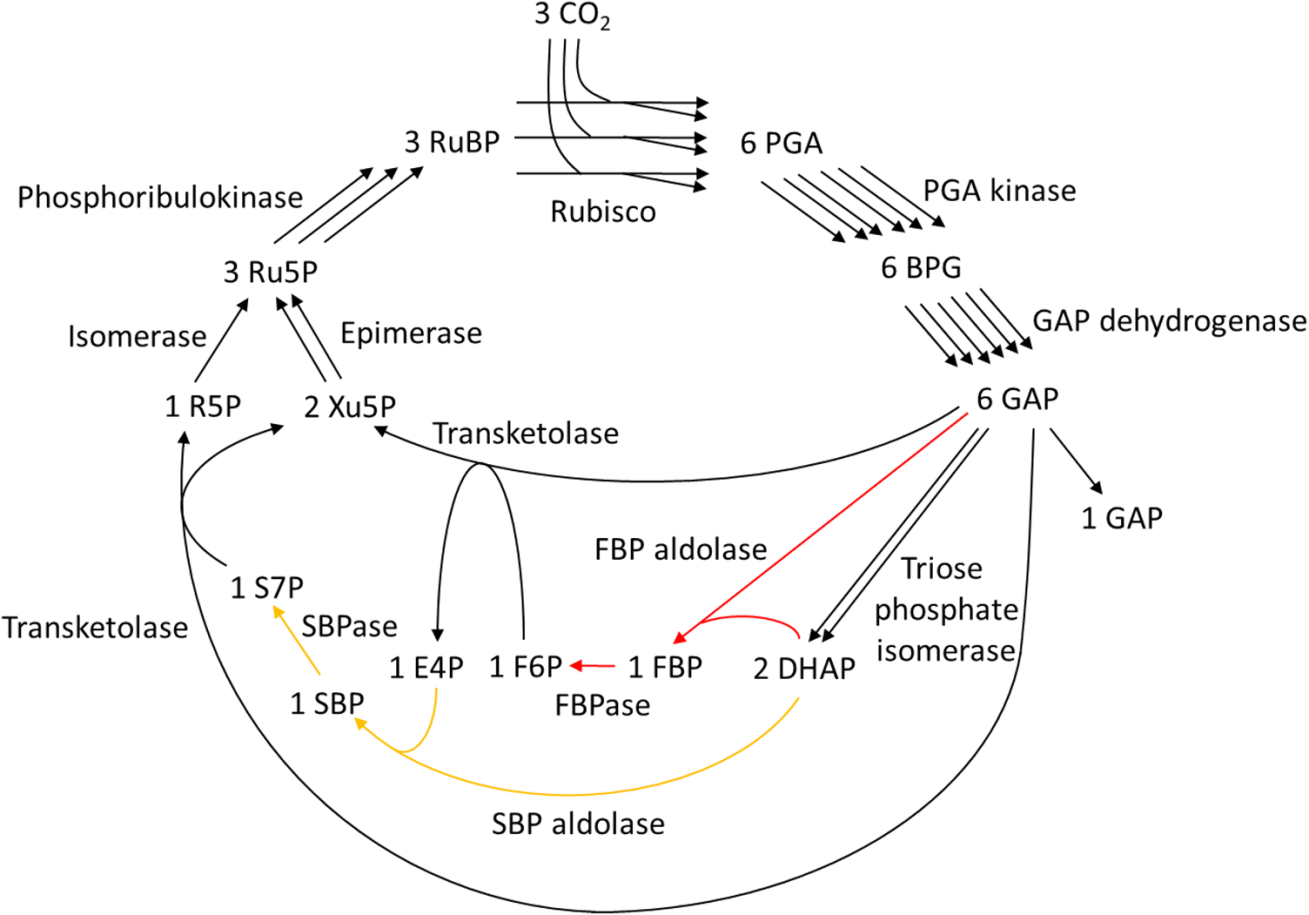
GAP formation from CO_2_ through the canonical Calvin–Benson cycle. The reactions corresponding to the red arrows appear in the S7P-forming transaldolase variant, but not in the S7P-removing transaldolase variant. The reactions corresponding to the orange arrows appear in the S7P-removing transaldolase variant, but not in the S7P-forming transaldolase variant. For abbreviations, the meaning of the numbers before each metabolite, and currency metabolites, please see the legend in Fig. 1. In this paper, the FBP and SBP formation reactions catalyzed by aldolase are named FBP aldolase and SBP aldolase, respectively.

**Figure 3:**
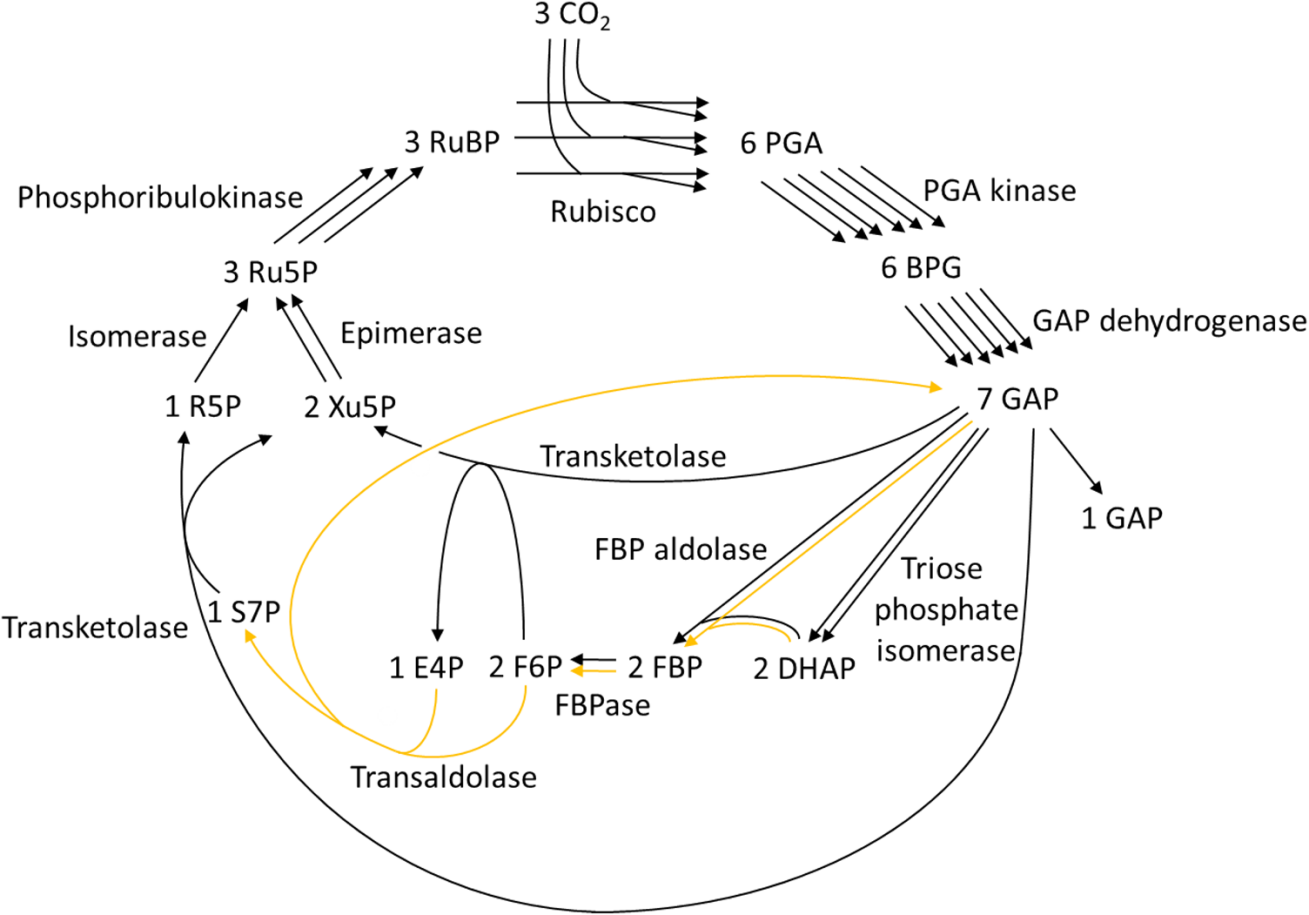
GAP formation from CO_2_ through the S7P-forming transaldolase variant of the Calvin–Benson cycle. The reactions corresponding to the orange arrows appear in neither the S7P-removing transaldolase variant nor the canonical Calvin–Benson cycle. For abbreviations, the meaning of the numbers before each metabolite, and currency metabolites, please see the legend in Fig. 1. In this paper, the FBP and SBP formation reactions catalyzed by aldolase are named FBP aldolase and SBP aldolase, respectively.

Table 2 shows the set of reactions to form GAP from CO_2_ constituting each of the canonical CB cycle, the S7P-forming transaldolase variant, and the S7P-removing transaldolase variant. In the present study, we performed computation by ExPA^15^ of elementary flux modes or extreme pathways^16,17^ in a biochemical reaction system consisting of import of CO_2_, export of GAP, the 13 reactions shown in Table 1, their reverse reactions, and transaldolase reactions in both directions. Values for the number of reactions per set shown in Table 2 were obtained from this computation. Please see Supplementary Information for details. Generally, any sets of indispensable reactions constituting a pathway from a specific source to a specific target are elementary flux modes or extreme pathways^16,17^. Thus, the novel S7P-removing transaldolase variant shown in Table 2 was obtained as an elementary flux mode or extreme pathway. In addition, the 2 known cycles of the canonical CB cycle and the S7P-forming transaldolase variant were also obtained as elementary flux mode or extreme pathway. Another calculation using the definition of reactions considering currency metabolites confirmed that each set of reactions shown in Table 2 results in the consumption of 3 molecules of CO_2_ accompanied by the production of one molecule of GAP with no difference in the balance of currency metabolites among the 3 sets. Figures 1, 2 and 3 show the S7P-removing transaldolase variant, the canonical CB cycle, and the S7P-forming transaldolase variant each functioning as the pathway for GAP formation from CO_2_, respectively.

**Table 2:** Set of reactions to form GAP from CO_2_ constituting each of the canonical CB cycle, the S7P-forming transaldolase variant and the S7P-removing transaldolase variant. GAP, glyceraldehyde 3-phosphate; CB cycle, Calvin-Benson cycle; S7P, sedoheptulose 7-phosphate^a^.

Reaction number and name		Variants including the canonical CB cycle, number of reactions per set		
		Canonical CB cycle	S7P-forming transaldolase variant	S7P-removing transaldolase variant
1	Rubisco	3	3	3
2	PGA kinase	6	6	6
3	GAP dehydrogenase	6	6	6
4	Triose phosphate isomerase	2	2	2
5	FBP aldolase	1	2	0
6	FBPase	1	2	0
7	Transketolase	1	1	1
8	SBP aldolase	1	0	2
9	SBPase	1	0	2
10	Transketolase	1	1	1
11	Isomerase	1	1	1
12	Epimerase	2	2	2
13	Phosphoribulokinase	3	3	3
14	S7P-forming transaldolase ^b^	0	1	0
15	S7P-removing transaldolase ^c^	0	0	1

^a^For other abbreviations, please see Table 1. The FBP and SBP formation reactions catalyzed by aldolase are named FBP aldolase and SBP aldolase, respectively.

^b^E4P + F6P → S7P + GAP.

^c^S7P + GAP → E4P + F6P.

## Discussion

Table 2 indicates that the canonical CB cycle, the S7P-forming transaldolase variant, and the S7P-removing transaldolase variant are different only in 6 reactions, which are FBP aldolase, FBPase, SBP aldolase, SBPase, S7P-forming transaldolase, and S7P-removing transaldolase. Please note that in this paper, the FBP and SBP formation reactions catalyzed by aldolase are named FBP aldolase and SBP aldolase, respectively. In the proposed S7P-removing transaldolase variant, transaldolase is used once in the S7P-removing direction, SBP aldolase reaction and SBPase reaction are involved twice each, but neither FBP aldolase reaction nor FBPase reaction. In the canonical CB cycle, transaldolase is not involved, but FBP aldolase reaction, FBPase reaction, SBP aldolase reaction, and SBPase reaction are involved once each. In the S7P-forming transaldolase variant, transaldolase is used once in the S7P-forming direction, and FBP aldolase reaction and FBPase reaction are involved twice each, but neither SBP aldolase reaction nor SBPase reaction.

Thus, whilst both the canonical CB cycle and the S7P-forming transaldolase variant require FBP aldolase reaction and FBPase reaction, the S7P-removing transaldolase variant requires neither FBP aldolase reaction nor FBPase reaction. Therefore, it is assumed that the S7P-removing transaldolase variant functions even if lacking FBPase, but neither the canonical CB cycle nor the S7P-forming transaldolase variant. The S7P-removing transaldolase variant explains photoautotrophic growth of an *Arabidopsis* mutant lacking FBPase^4^ most easily among the 3 cycles, whilst it is independent from a mechanism assuming a cytosolic path bypassing chloroplastic FBPase reaction, of increased cyclic photosynthetic electron flow in the mutant^2^.

In addition, the S7P-removing transaldolase variant as well as the canonical CB cycle is dependent on SBPase, but the S7P-forming transaldolase variant is not. SBPase is a critical enzyme for photosynthesis and the target of manipulation for enhancing photosynthetic carbon metabolism^2,18–25^, the mechanism of which can be explained by dependency on SBPase for the S7P-removing transaldolase variant and the canonical CB cycle, but not for the S7P-forming transaldolase variant.

Although studies had indicated that transaldolase is present in chloroplasts^6,7^, the chloroplastic compartmentation of transaldolase was recognized to be controversial based on the reported dependency of photosynthesis on SBPase^26^. This was because no variants dependent on both transaldolase and SBPase were known. However, recently *gsm2*, a glucose-hypersensitive *Arabidopsis* mutant with a notably chlorotic-cotyledon phenotype, has been isolated, and GSM2 encoded by *AT5G13420* has been reported to be a chloroplast-localized transaldolase, which was deduced from the transaldolase activity in *gsm2* seedlings and colocalization of enhanced green fluorescent protein fused to GSM2 and chlorophyll^12^. Together with this recent evidence, the S7P-removing transaldolase variant of the CB cycle, which is dependent on both transaldolase and SBPase, would provide new insights into the chloroplastic compartmentation of transaldolase. The S7P-removing transaldolase variant clearly indicates that SBPase and transaldolase can collaborate with each other in photosynthetic carbon metabolism. Whether co-overexpression of SBPase and transaldolase increases photosynthetic carbon metabolism should be investigated.

Whilst only the S7P-removing transaldolase variant can function in the absence of FBPase among the canonical CB cycle, the S7P-forming transaldolase variant, and the S7P-removing transaldolase variant, the S7P-removing transaldolase variant can function only in the presence of transaldolase. Therefore, photoautotrophic growth of mutants lacking FBPase in photosynthetic species expressing transaldolase in chloroplasts, which is supposed to be due to the S7P-removing transaldolase variant, is predicted to be reduced by knockout or knockdown of transaldolase.

Figures 1, 2 and 3 indicate that the S7P-removing transaldolase variant as well as the 2 known cycles of the canonical CB cycle and the S7P-forming transaldolase variant constitutes a cycle to regenerate RuBP. Two small cycles, F6P-E4P-SBP-S7P-F6P and E4P-SBP-S7P-E4P, are found in the S7P-removing transaldolase variant shown in Fig. 1. They do not pass through RuBP. As shown in Figs. 2 and 3, none of the canonical CB cycle and the S7P-forming transaldolase variant have such small cycles. These 2 small cycles make the pathway structure of the S7P-removing transaldolase variant complicated. Such complexity of the pathway structure may have delayed awareness of the S7P-removing transaldolase variant.

In the canonical CB cycle and the S7P-forming transaldolase variant shown in Figs. 2 and 3, FBP is inevitably formed from CO_2_ and located upstream of S7P^5,14^. On the other hand, FBP does not exist in the S7P-removing transaldolase variant shown in Fig. 1, where F6P is located downstream of S7P and GAP. To explain the finding^3^ that treatment of an illuminated *Arabidopsis* rosette with ^13^CO_2_ resulted in slower labeling of FBP than S7P, Szecowka et al. speculated that a significant part of the FBP pool is not directly involved in the CB cycle^3^. The S7P-removing transaldolase variant matches this speculation and is more consistent with the time course of FBP and S7P labeling stated than the S7P-forming transaldolase variant and the canonical CB cycle.

Now, to theoretically evaluate the rate of metabolism through the S7P-removing transaldolase variant, we consider a system that mimics one chloroplast in which *c* mol of GAP is produced from 3*c* mol of CO_2_ in 1 s. Here we take the position to believe that GAP is generated from CO_2_ by the canonical CB cycle. This means that each reaction of the canonical CB cycle must occur in this system at a rate (mol/s) equal to *c* times the value of the column for the canonical CB cycle in Table 2. In addition, we assume that transaldolase is present in this system and that the S7P-forming transaldolase reaction and the S7P-removing transaldolase reaction each occur at a rate equal to *c* (mol/s), where the S7P-forming transaldolase reaction and the S7-removing transaldolase reaction are set to occur at the same rate so as not to affect the concentration of metabolites.

The only reactions that metabolize E4P present in this system are the SBP aldolase reaction and the S7P-forming transaldolase reaction. The rate of the SBP aldolase reaction and the rate of the S7P-forming transaldolase reaction are both equal to *c*. Therefore, the probability that E4P is metabolized by the SBP aldolase reaction and the probability that it is metabolized by the S7P-forming transaldolase reaction are both 0.5, and are not affected by the E4P concentration. In addition, there is no reaction that metabolizes S7P existing in this system other than the transketolase reaction that produces R5P and the S7P-removing transaldolase reaction. The rate of the transketolase reaction that produces R5P and the rate of the S7P-removing transaldolase reaction are both equal to *c*. Therefore, the probability that S7P is metabolized by the transketolase reaction that produces R5P and the probability that it is metabolized by the S7P-removing transaldolase reaction are both 0.5, and are not affected by the S7P concentration. Therefore, the following A, B, and C hold.

(A) The probability that E4P is metabolized by the SBP aldolase reaction and S7P is metabolized only by the transketolase reaction that produces R5P (not metabolized by the S7P-removing transaldolase reaction) is equal to 0.25 (0.5 squared). This only happens with the canonical CB cycle.
(B) The probability that E4P is metabolized by the S7P-forming transaldolase reaction and S7P is metabolized only by the transketolase reaction that produces R5P (not metabolized by the S7P-removing transaldolase reaction) is equal to 0.25 (0.5 squared). This only happens with the S7P-forming transaldolase variant. In this simulation, half of the S7P produced by the S7P-forming transaldolase reaction is metabolized by the transketolase reaction that produces R5P, and the other half is returned by the S7P-removing transaldolase reaction (reverse reaction of the S7P-forming transaldolase reaction).
(C) The probability that E4P is metabolized by the SBP aldolase reaction and S7P is metabolized by the S7P-removing transaldolase reaction is equal to 0.25 (0.5 squared). This only happens with the S7P-removing transaldolase variant. In this simulation, half of the E4P produced by the S7P-removing transaldolase reaction is metabolized by the SBP aldolase reaction, and the other half is returned by the S7P-forming transaldolase reaction (reverse reaction of the S7P-removing transaldolase reaction).

That is, each of the canonical CB cycle, the S7P-forming transaldolase variant, and the S7P-removing transaldolase variant occurs with a 1/4 probability. The remaining quarter is the sum of the probabilities of GAP-producing pathways that include any number of interconversions between S7P + GAP and E4P + F6P by the S7P-forming transaldolase reaction and the S7P-removing transaldolase reaction.

Similarly, if it is assumed that the S7P-forming transaldolase reaction and the S7P-removing transaldolase reaction each occur at a rate equal to *k* (mol/s) instead of *c* (mol/s), the canonical CBC cycle occurs with a probability of *c*^2^ ÷ (*c* + *k*)^2^, and the S7P-forming transaldolase variant and the S7P-removing transaldolase variant each occur with a probability of *c* × *k* ÷ (*c* + *k*)^2^. This indicates that in the presence of transaldolase in chloroplasts, the S7P-forming transaldolase variant and the S7P-removing transaldolase variant can function substantially depending on transaldolase activity, in addition to the canonical CB cycle.

In chloroplasts without transaldolase, only the canonical CB cycle is functional among the canonical CB cycle, the S7P-forming transaldolase variant, and the S7P-removing transaldolase variant. There, photosynthetic carbon metabolism ceases when either SBPase or FBPase is lost. On the other hand, in chloroplasts in which transaldolase is present, the canonical CB cycle, the S7P-forming transaldolase variant, and the S7P-removing transaldolase variant are all functional. There, photosynthetic carbon metabolism by the S7P-forming transaldolase variant or the S7P-removing transaldolase variant continues even if either FBPase or SBPase is lost. Thus, the presence of transaldolase in chloroplasts makes photosynthetic carbon metabolism in chloroplasts robust. The S7P-removing transaldolase variant contributes to this robustness.

Which pathway among the canonical CB cycle, the S7P-forming transaldolase variant, and the S7P-removing transaldolase variant functions predominantly would depend on the availability of FBPase, SBPase, and transaldolase. *Arabidopsis* mutants lacking only one of these 3 enzymes are known. As for FBPase, as mentioned above, an *Arabidopsis* mutant lacking FBPase has been isolated and found to grow in soil to some extent^4^, which can be explained by the S7P-removing transaldolase variant. As for SBPase, an *Arabidopsis* mutant, *sbp*, has been isolated as a mutant lacking SBPase^27^. Although the CO_2_ assimilation rate of the fully expanded leaves detached from *sbp* mutant plants showed a negative value, the *sbp* mutant plants could survive without SBPase^27^. To explain the survival of *sbp* mutant plants, one possibility was suggested that FBPase might be able to catalyze the conversion of SBP to S7P. However, the S7P-transaldolase variant can explain the survival of *sbp* mutant plants, even assuming no conversion of SBP to S7P. As for transaldolase, as mentioned above, a glucose-hypersensitive *Arabidopsis* mutant, *gsm2*, has been isolated as a mutant lacking chloroplast-localized transaldolase^12^. The growth of the *gsm2* mutant can be explained by the canonical CB cycle. Thus, the growth or survival of *Arabidopsis* mutants lacking only one of FBPase, SBPase, and transaldolase can be understood as an example of the robustness found in the system consisting of the canonical CB cycle, the S7P-forming transaldolase variant, and the S7P-removing transaldolase variant. Whilst the difference in growth between the wild type and the mutant lacking either FBPase or SBPase was prominent^4,27^, the growth of the *gsm2* mutant lacking transaldolase under sugar-free conditions was similar to that of the wild type^12^. In the *gsm2* mutant, dysfunction of the S7P-forming transaldolase variant and the S7P-removing transaldolase variant should occur due to the absence of GSM2, a transaldolase. This dysfunction might be compensated for by upregulation of the canonical CB cycle. On the other hand, in mutants lacking either FBPase or SBPase, dysfunction of the canonical CB cycle should occur. This dysfunction does not seem to be fully compensated for by upregulation of the S7P-froming transaldolase variant or the S7P-removing transaldolase variant, suggesting that the transaldolase required for the 2 transaldolase variants is present rather than in excess and may function in a substrate concentration-dependent manner rather than being actively regulated.

The canonical CB cycle is recognized as a redox regulated process^28^. FBPase in chloroplasts is a reductively-activated regulatory enzyme^28^. SBPase is also a reductively-activated regulatory enzyme^28^ and is reported to be susceptible to oxidative damage^27^. GSM2, a transaldolase, has also been shown to contribute to reactive oxygen species homeostasis in *Arabidopsis*, where it is assumed to catalyze the transaldolase reaction in the S7P-removing direction to produce E4P, one of the initial precursors of the shikimate pathway^12^. There is a possibility that transaldolase might function to keep FBPase and SBPase activated reductively and to enhance the protection of SBPase from oxidative damage. This possibility means that transaldolase sustains photosynthetic carbon metabolism by contributing to reactive oxygen species homeostasis and by contributing to the above robustness through the S7P-forming transaldolase variant and the S7P-removing transaldolase variant. The contribution of transaldolase to reactive oxygen species homeostasis, if it is through E4P production by the S7P-removing transaldolase reaction, would be more collaborative with the S7P-removing transaldolase variant than with the S7P-forming transaldolase variant.

Proteomic analysis of chloroplasts from *Nicotiana tabacum*^29^ has shown that proteins assumed to be transaldolases are present in chloroplasts. The proteins encoded by *TAL1* and *TAL2* from *Chlamydomonas reinhardtii* are assumed to be transaldolases^8^. Proteomic analysis of chloroplasts from *Chlamydomonas reinhardtii*^30^ has shown that both of the proteins encoded by *TAL1* and *TAL2* exist in chloroplasts and that the protein encoded by *TAL2* is more abundant than the protein encoded by *TAL1*. These suggest that the S7P-removing transaldolase variant functions in *Nicotiana tabacum* and *Chlamydomonas reinhardtii*. It has been reported that overexpression of a cyanobacterial fructose-1,6-/sedoheptulose-1,7-bisphosphatase (FBP/SBPase) in tobacco enhances photosynthesis and growth^31^. On the other hand, it has been reported that overexpression of FBPase in *Chlamydomonas reinhardtii* has a negative effect on growth^32^. These phenomena indicate that overexpression of FBPase has a different effect on growth depending on the level of SBPase activity or species-related factors. The S7P-removing transaldolase variant neither explains them directly nor is related to them apparently. In addition, it is not regulated through FBPase activity. These clearly show that photosynthetic carbon metabolism cannot be explained by only the S7P-removing transaldolase variant. However, if the canonical CB cycle and transaldolase coexist in the chloroplast, the S7P-removing transaldolase variant inevitably functions as above. Therefore, the S7P-removing transaldolase variant should be considered as one of the important mechanisms or pathways to explain photosynthetic carbon metabolism. This is also true for the S7P-forming transaldolase variant.

The CB cycle is understood to function in Cyanobacteria^33^. FBP/SBPase has been isolated from Cyanobacteria^34^. The cyanobacterial FBP/SBPase, which hydrolyzes both FBP and SBP with almost equal specific activities, can contribute to photosynthetic carbon metabolism in Cyanobacteria. For cyanobacterial proteins assumed to be transalodolases, UniProtKB has more than 800 entries having the value ‘Protein inferred by homology’, which are from more than 600 strains^8^. These suggest that the S7P-removing transaldolase variant functions in Cyanobacteria.

In conclusion, the S7P-removing transaldolase variant would well explain the dependency of photosynthetic carbon metabolism on SBPase and the FBP- or FBPase-related findings of photosynthetic carbon metabolism difficult to explain by the canonical CB cycle or the S7P-forming transaldolase. The S7P-removing transaldolase variant deserves further investigation.

## Methods

Computation of elementary flux modes or extreme pathways to find all pathways to form GAP from CO_2_ was performed by ExPA^15^. Any elementary flux mode or extreme pathway corresponding to a pathway to form GAP from CO_2_ was obtained as a set of reactions to form GAP from CO_2_, giving the number of reactions for each reaction name in that set. The pathway structure corresponding to such a set of reactions to form GAP from CO_2_ was manually drawn. When drawing each pathway structure, first the names of the metabolites on the pathway were placed so that the same name would appear only once. Then, they were connected by arrows, each belonging to one reaction, where the number of arrows reflects the number of reactions included in the set of reactions. Then, one arrow and one GAP corresponding to the formation of the final product, GAP, were placed. For each metabolite except the initial source CO_2_ and the final product GAP, the number of arrows entering the metabolite was the same as the number of arrows exiting the metabolite. Finally, a number was written before each metabolite so that it was equal to the number of arrows entering or exiting the metabolite.

## Supplementary Information

Supplementary Information.
