# Supplementary material for: A novel variant of the Calvin–Benson cycle bypassing fructose bisphosphate
Source: Sci Rep. 2022 Mar 16;12:3984. doi: 10.1038/s41598-022-07836-7 (PMC8927339; doi:10.1038/s41598-022-07836-7)
Supplement: Supplementary file 1 — Supplementary Information. [file 41598_2022_7836_MOESM1_ESM.pdf]

Procedures to compute elementary flux modes or extreme pathways forming glyceraldehyde 3-phosphate (GAP) from CO_2

Please prepare the 3 text files named Paths_example.txt, perform_expa.bat, and CO2_to_GAP.expa as described below. The following procedure was successful on a Windows PC on August 18, 2021.

1. Go to https://systemsbiology.ucsd.edu/Downloads/ExtremePathwayAnalysis
2. Get expa.zip by clicking “A new ExPA program (02/16/05)”.
3. Extract expa.exe from expa.zip.
4. Make a working directory and put the 3 files of expa.exe, perform_expa.bat, and CO2_to_GAP.expa there.
5. Double-click on perform_expa.bat in the directory from Windows Explorer.
6. Find Paths.txt generated in the same directory. The content of Paths.txt must be the same as that of Paths_example.txt. Paths.txt as well as Paths_example.txt carries a matrix, where each of rows corresponds to one elementary flux mode or extreme pathway and each of columns to one of the fluxes defined in CO2_to_GAP.expa. The last 3 rows of the matrix correspond to the 3 columns in Table 2, which correspond to the S7P-removing transaldolase variant, the S7P-forming transaldolase variant, and the canonical Calvin-Benson cycle. The other rows correspond to pathways neither consuming CO_2 nor producing GAP. They include 2 cycles composed of 5 reactions, but those cycles require the unrealistic reverse reaction of FBPase or SBPase. Each row of CO2_to_GAP.expa beginning at ‘>’ corresponds to the reaction with the same name as defined in Table 1, whilst each row of CO2_to_GAP.expa beginning at <’ corresponds to the reverse reaction of the reaction with the same name as defined in Table 1.

Paths_example.txt

0	0	0	0	0	0	0	1	1	0	0	0	0	0	0	0	0	0	1	1	0	0	0	0	0	0	0	1	0	0
0	0	0	0	1	1	0	0	0	0	0	0	0	1	0	0	0	0	0	0	0	1	1	0	0	0	0	0	0	0
0	0	0	0	0	0	0	0	0	0	0	0	0	1	0	0	0	0	0	0	0	0	0	0	0	0	0	1	0	0
0	0	0	0	0	0	1	0	0	0	0	0	0	0	0	0	0	0	0	0	1	0	0	0	0	0	0	0	0	0
0	0	0	0	0	0	0	0	0	0	1	0	0	0	0	0	0	0	0	0	0	0	0	0	1	0	0	0	0	0
0	0	0	0	0	0	0	0	0	1	0	0	0	0	0	0	0	0	0	0	0	0	0	1	0	0	0	0	0	0
0	0	0	0	0	1	0	0	0	0	0	0	0	0	0	0	0	0	0	1	0	0	0	0	0	0	0	0	0	0
0	0	0	0	1	0	0	0	0	0	0	0	0	0	0	0	0	0	1	0	0	0	0	0	0	0	0	0	0	0
0	1	0	0	0	0	0	0	0	0	0	0	0	0	0	1	0	0	0	0	0	0	0	0	0	0	0	0	0	0
1	0	0	0	0	0	0	0	0	0	0	0	0	0	1	0	0	0	0	0	0	0	0	0	0	0	0	0	0	0
0	0	0	0	0	0	0	0	0	0	0	0	1	0	0	0	0	0	0	0	0	0	0	0	0	0	1	0	0	0
0	0	1	0	0	0	0	0	0	0	0	0	0	0	0	0	1	0	0	0	0	0	0	0	0	0	0	0	0	0
0	0	0	0	0	0	0	0	1	0	0	0	0	0	0	0	0	0	0	0	0	0	1	0	0	0	0	0	0	0
0	0	0	0	0	0	0	1	0	0	0	0	0	0	0	0	0	0	0	0	0	1	0	0	0	0	0	0	0	0
0	0	0	0	0	0	0	0	0	0	0	1	0	0	0	0	0	0	0	0	0	0	0	0	0	1	0	0	0	0
0	0	0	1	0	0	0	0	0	0	0	0	0	0	0	0	0	1	0	0	0	0	0	0	0	0	0	0	0	0
3	6	6	2	0	0	1	2	2	1	1	2	3	0	0	0	0	0	0	0	0	0	0	0	0	0	0	1	-3	1
3	6	6	2	2	2	1	0	0	1	1	2	3	1	0	0	0	0	0	0	0	0	0	0	0	0	0	0	-3	1
3	6	6	2	1	1	1	1	1	1	1	2	3	0	0	0	0	0	0	0	0	0	0	0	0	0	0	0	-3	1

perform_expa.bat

```
expa -v CO2_to_GAP.expa
```

CO2_to_GAP.expa

```
(Internal Fluxes)
>Rubisco                        I    -1 CO2  -1 RuBP    2 PGA
>PGA_kinase                     I    -1 PGA             1 BPG
>GAP_dehydrogenase              I    -1 BPG             1 GAP
>Triose_phosphate_isomerase     I    -1 GAP             1 DHAP
>FBP_aldolase                   I    -1 GAP  -1 DHAP    1 FBP
>FBPase                         I    -1 FBP             1 F6P
>Transketolase                  I    -1 F6P  -1 GAP     1 E4P   1 Xu5P
>SBP_aldolase                   I    -1 E4P  -1 DHAP    1 SBP
>SBPase                         I    -1 SBP             1 S7P
>Transketolase                  I    -1 S7P  -1 GAP     1 R5P   1 Xu5P
>Isomerase                      I    -1 R5P             1 Ru5P
>Epimerase                      I    -1 Xu5P            1 Ru5P
>Phosphoribulokinase            I    -1 Ru5P            1 RuBP
S7P-forming_transaldolase       I    -1 E4P  -1 F6P     1 S7P   1 GAP
<Rubisco                        I     1 CO2   1 RuBP   -2 PGA
<PGA_kinase                     I     1 PGA            -1 BPG
<GAP_dehydrogenase              I     1 BPG            -1 GAP
<Triose_phosphate_isomerase     I     1 GAP            -1 DHAP
<FBP_aldolase                   I     1 GAP   1 DHAP   -1 FBP
<FBPase                         I     1 FBP            -1 F6P
<Transketolase                  I     1 F6P   1 GAP    -1 E4P  -1 Xu5P
<SBP_aldolase                   I     1 E4P   1 DHAP   -1 SBP
<SBPase                         I     1 SBP            -1 S7P
<Transketolase                  I     1 S7P   1 GAP    -1 R5P  -1 Xu5P
<Isomerase                      I     1 R5P            -1 Ru5P
<Epimerase                      I     1 Xu5P           -1 Ru5P
<Phosphoribulokinase            I     1 Ru5P           -1 RuBP
S7P-removing_transaldolase      I     1 E4P   1 F6P    -1 S7P  -1 GAP
(Exchange Fluxes)
CO2                             Input
GAP                             Output
```
